# Surface glycosaminoglycans mediate adherence between HeLa cells and *Lactobacillus salivarius* Lv72

**DOI:** 10.1186/1471-2180-13-210

**Published:** 2013-09-17

**Authors:** Rebeca Martín, Carla Martín, Susana Escobedo, Juan E Suárez, Luis M Quirós

**Affiliations:** 1 Área de Microbiología, Universidad de Oviedo, Julián Clavería 6 33006 Oviedo, Spain; 2 Instituto Universitario de Biotecnología, Universidad de Oviedo, Oviedo, Spain; 3 Instituto de Productos Lacteos de Asturias (IPLA-CSIC), Villaviciosa, Spain; 4 Instituto Univesitario de Oncología del Principado de Asturias (IUOPA), Universidad de Oviedo, Oviedo, Spain

**Keywords:** Proteoglycans, Glycosaminoglycans, Vaginal *Lactobacillus*, Epithelial cell cultures

## Abstract

**Background:**

The adhesion of lactobacilli to the vaginal surface is of paramount importance to develop their probiotic functions. For this reason, the role of HeLa cell surface proteoglycans in the attachment of *Lactobacillus salivarius* Lv72, a mutualistic strain of vaginal origin, was investigated.

**Results:**

Incubation of cultures with a variety of glycosaminoglycans (chondroitin sulfate A and C, heparin and heparan sulfate) resulted in marked binding interference. However, no single glycosaminoglycan was able to completely abolish cell binding, the sum of all having an additive effect that suggests cooperation between them and recognition of specific adhesins on the bacterial surface. In contrast, chondroitin sulfate B enhanced cell to cell attachment, showing the relevance of the stereochemistry of the uronic acid and the sulfation pattern on binding. Elimination of the HeLa surface glycosaminoglycans with lyases also resulted in severe adherence impairment. Advantage was taken of the *Lactobacillus*-glycosaminoglycans interaction to identify an adhesin from the bacterial surface. This protein, identify as a soluble binding protein of an ABC transporter system (OppA) by MALDI-TOF/(MS), was overproduced in *Escherichia coli*, purified and shown to interfere with *L. salivarius* Lv72 adhesion to HeLa cells.

**Conclusions:**

These data suggest that glycosaminoglycans play a fundamental role in attachment of mutualistic bacteria to the epithelium that lines the cavities where the normal microbiota thrives, OppA being a bacterial adhesin involved in the process.

## Background

The vaginal microbiota of healthy women of reproductive age is dominated by lactobacilli. Their proportion in this habitat is consistently higher than 70%, in some cases being practically exclusive [1-3]. The evidence compiled about the mutualistic role of lactobacilli on the mucous membranes, together with their harmlessness, has promoted their use as probiotic agents [4]. Isolates obtained from feces, vaginal exudates and even mucous membranes resulting from surgery, have been tested for epithelial adherence, acid, bacteriocin and H_2_O_2_ production and lack of transmissible antibiotic resistances with the aim of understanding the basis of their interaction with the mucosal surfaces and, ultimately, using them as probiotic agents [1,2].

Adherence to the epithelium of the cavity to be colonized is of paramount importance to compete with colonization by potential pathogens and to avoid sweeping by the circulating fluids. Impairment of adherence by treatment of microbial or epithelial cells with proteases, lipases or periodic acid suggested that the bacterial adhesins and cellular receptors are proteins, lipids or polysaccharides respectively [5-8]. Furthermore, identification of the proteins secreted by the bacteria and those anchored to its cell wall has provided lists of polypeptides putatively involved in mucous adherence. Curiously, this approach has identified enzymes related to sugar catabolism, such as glyceraldehyde-3-phosphate dehydrogenase and enolase [9-12].

Cellular receptors that bind bacteria have to be both ubiquitous on the surface of the epithelial cells while showing enough variability as to account for the observed organotropism shown. These conditions are met by proteoglycans (PGs), which are made up of specific protein cores covalently bound to linear polysaccharides named glycosaminoglycans (GAGs). The GAGs are built of repeat disaccharide subunits, whose composition allows their classification into different groups: i) heparin/heparan sulphate (HS), containing glucuronic acid (GlcA) and N-acetyl glucosamine (GlcNAc); ii) chondroitin/dermatan sulphate (CS/DS), where GlcA is replaced by N-acetylgalactosamine (GalNAc); iii) keratan sulphate, with galactose and GlcNAc, and iv) hyaluronic acid (HA), with the same disaccharide unit as HS, but unmodified and devoid of the protein stem.

During their biosynthesis, all GAGs but HA undergo different modification reactions that can involve N-deacetylations, epimerizations and various O-sulfations. The structure of the GAG chains expressed is regulated and dynamically adapted. To perform this task, multiple isoenzymes can perform the catalysis [13-15]. Each isoenzyme shows particular substrate specificity, and their expression vary depending on the cells, the tissues, the state of development and the physiological and pathological conditions.

A variety of functions have been ascribed to PGs, including cell adhesion and migration, organization of the cytoskeleton and of the extracelullar matrix (ECM), regulation of proliferation, differentiation and morphogenesis, and tissue repair and inflammation [16-18]. Furthermore, they act as co-receptors for multiple soluble ligands including cytokines, chemokines, growth factors, enzymes and enzyme inhibitors, thus collaborating in intercellular communication and tissue differentiation [16,19,20].

PGs in general and especially those with heparan sulphate (HSPGs) have been proposed as the receptors that facilitate recognition and binding between eukaryotic membranes and microbial pathogens, including bacteria, viruses and protozoa. Their role as receptors for *Neisseria meningitidis*[21], *N. gonorrhoeae*[22,23], *Mycobacterium tuberculosis*[24], *Enterococcus faecalis*[25], *Listeria monocytogenes*[26], *Streptococcus* and *Staphylococcus*[27], *Brucella*[28], *Escherichia coli*[29] and even intracellular parasites such as *Chlamidia pneumoniae*[30] have been described. Besides this, it seems that binding of group A streptococci to GAGs leads to a cytoskeleton conformational change that allows pathogen penetration [31,32]. The requirement of GAGs for viral infection has been demonstrated, among others, for papilloma virus [33], herpes virus [34], and HIV [35]. Finally, it is known that GAGs act as receptors for *Toxoplasma gondii*[36], *Leishmania*[37] and *Plasmodium*[38]. However, the microbial ligands involved in most of these processes have not yet been identified. This role of PGs as the eukaryotic receptors for many pathogens is the basis of our initial hypothesis which suggests the same function of these molecules when interacting with autochthonous no pathogenic microorganisms such as lactobacilli.

In this report we provide data on the involvement of GAGs in attachment of *Lactobacillus salivarius* Lv72, isolated from a human vaginal exudate, to cultures of HeLa cells. Based on these data, a bacterial adhesin was identified which, once purified, significantly interfered with attachment of the lactobacilli to HeLa cell cultures.

## Results

### Interference of GAGs on HeLa cell-*Lactobacillus salivarius* Lv72 adhesion

**Figure 1:**
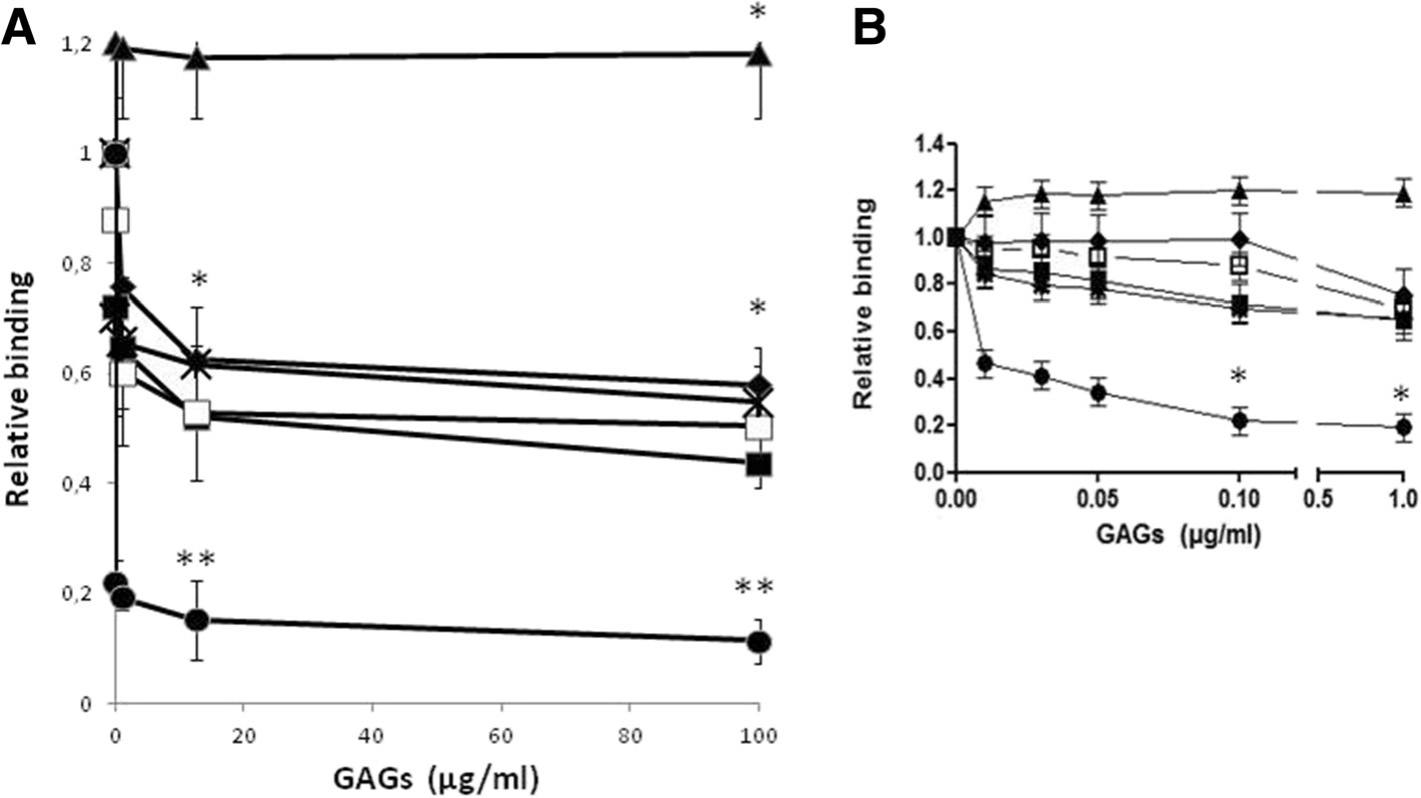
**Inhibition of *Lactobacillus *attachment to HeLa cells by the presence of different GAGs. A)** Adhesion of *Lactobacillus salivarius* Lv72 to HeLa cells co-incubated in the presence of different concentrations of heparin (♦), HS (_*_), CS A (■), CS C (□), CS B (▲) and a mixture of all GAGs except CS B (final concentration) (●). **B)** Detail of the inhibitory effect at concentrations below 1 μg /ml. n=9 ANOVA test **, p-value < 0.001; *, p-value < 0.05 vs adhesion of *Lactobacillus salivarius* Lv72 to HeLa cells without interferences.

To study the role of GAGs on Lv72 adhesion to HeLa cells, addition of commercial preparations of HS, heparin, CS A or CS C to HeLa to cell monolayers was performed immediately before the addition of exponentially growing *L. salivarius* Lv72 cells. The results showed a decrease in the adherence between them (Figure 1). This depletion, although being dose dependent, does not follow a linear correlation. The estimated dissociation constants (K_D_) were of 2.5 nM for HS, 6.8 nM for CS A, 39.9 nM for CS C and 280.9 nM for heparin, which indicates that the affinity of the bacteria for the different receptors varied markedly, up to two orders of magnitude between HS and heparin. However, care must be taken with this interpretation, as the K_D_s are approximate values. Surprisingly, CS B did not produce any inhibitory effect, and even promoted a slight increase in the adhesion (Figure 1). Remarkably, the combined use of these GAGs dramatically increased the inhibition, reaching values up to 85% and 90% at total concentrations of 10 and 100 μg/ml respectively, although this effect was not strictly additive (Figure 1A).

### Effect of cell surface GAGs digestion on adherence

**Figure 2:**
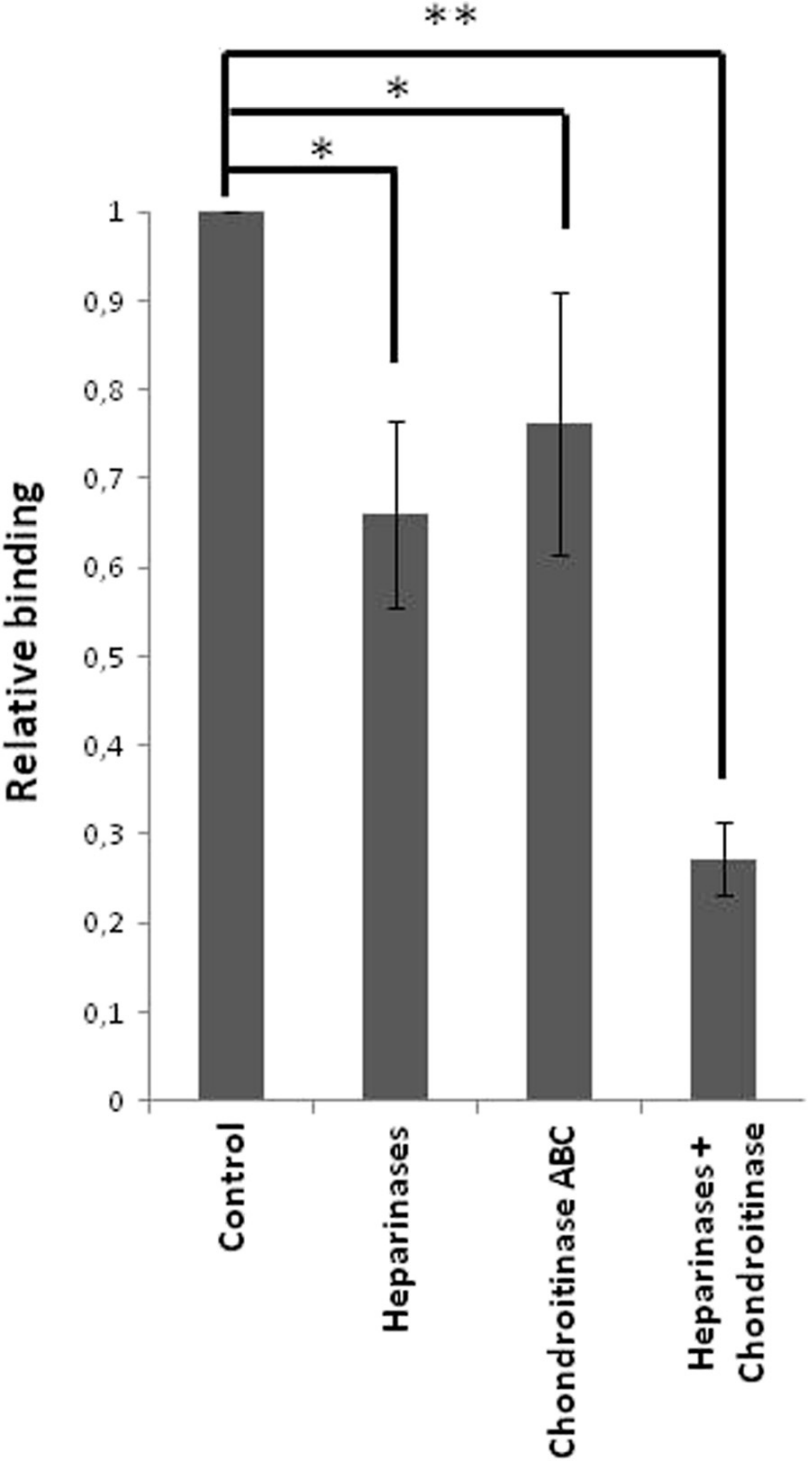
**Effect of the pre-treatment of HeLa cell cultures with GAG lyases on attachment of *L. salivarius *Lv72.** HeLa cells were treated with heparinases, chondroitinase ABC or heparinises + chondroitinase ABC before the co-incubation with the lactobacilli. n=9 ANOVA test **, p-value < 0.001; *, p-value < 0.05.

To investigate further the adherence of Lv72 to the GAGs, cell surface GAGs were removed by digestion with bacterial lyases, and the effect of this treatment on the binding of the bacteria was determined. Treatment with chondroitinase ABC, which degrades the three CS variants, resulted in reduced binding (Figure 2), slightly lower than that observed for high concentrations of the GAGs in the competition experiment. Furthermore, the concurrent degradation of heparan sulfate with heparinase I, which cleaves at the linkages between hexosamines and O-sulfated iduronic acids, heparinase III, which cleaves at the linkages between hexosamine and glucuronic acid, and heparinase II, which cleaves with lower selectivity linkages between hexosamines and uronic acid residues (both glucuronic and iduronic), resulted in a decrease in binding comparable to that obtained in competition experiments (Figure 2). Moreover, the simultaneous degradation with chondroitinase and heparinases produced an additive effect that reduced the binding of the bacteria (Figure 2).

### Differential effect of GAGs obtained from different cell types on adherence interference

**Figure 3:**
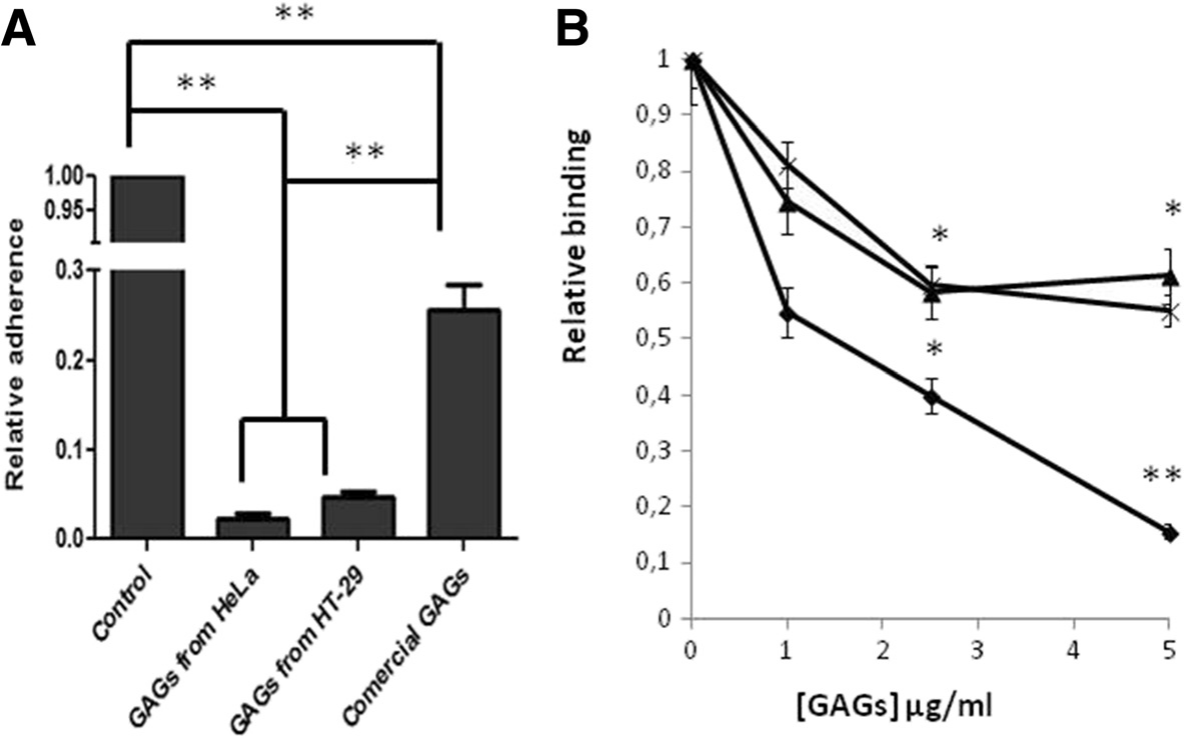
**Inhibition of *L. salivarius *attachment to HeLa cells by the presence of GAGs of different origins. A)** Relative adherence of the lactobacilli to HeLa cells co-incubated in the presence of 100 μg/ml of total GAGs extracted from HeLa and HT-29 cells and from commercially available, heterologous sources; n=9 ANOVA test **, p-value < 0.001; *, p-value < 0.05. **B)** Adhesion of *L. salivarius* Lv72 to HeLa cells co-incubated in the presence of increasing concentrations of HS (X), CS (▲) and a mixture of both (♦) extracted from HeLa cell cultures, n=9 ANOVA test **, p-value < 0.001; *, p-value < 0.05 vs adhesion of *Lactobacillus salivarius* Lv72 to HeLa cells without interferences.

To study the influence of the cellular type, GAGs were extracted from HeLa and HT-29 cell cultures and used in adherence assays. The results showed that the molecules isolated from human epithelial cells inhibited the binding of the lactobacilli more efficiently than commercially available GAGs, from pig or beef tissues (Figure 3A). GAGs from HT-29 and HeLa cultures were three and ten times more effective than the heterologous ones. Finally, soluble HS and CS purified from HeLa cells have similar effects on the adhesion of *L. salivarius* Lv 72 to HeLa cells (Figure 3B).

### Identification and testing of bacterial adhesins able to bind GAGs

**Figure 4:**
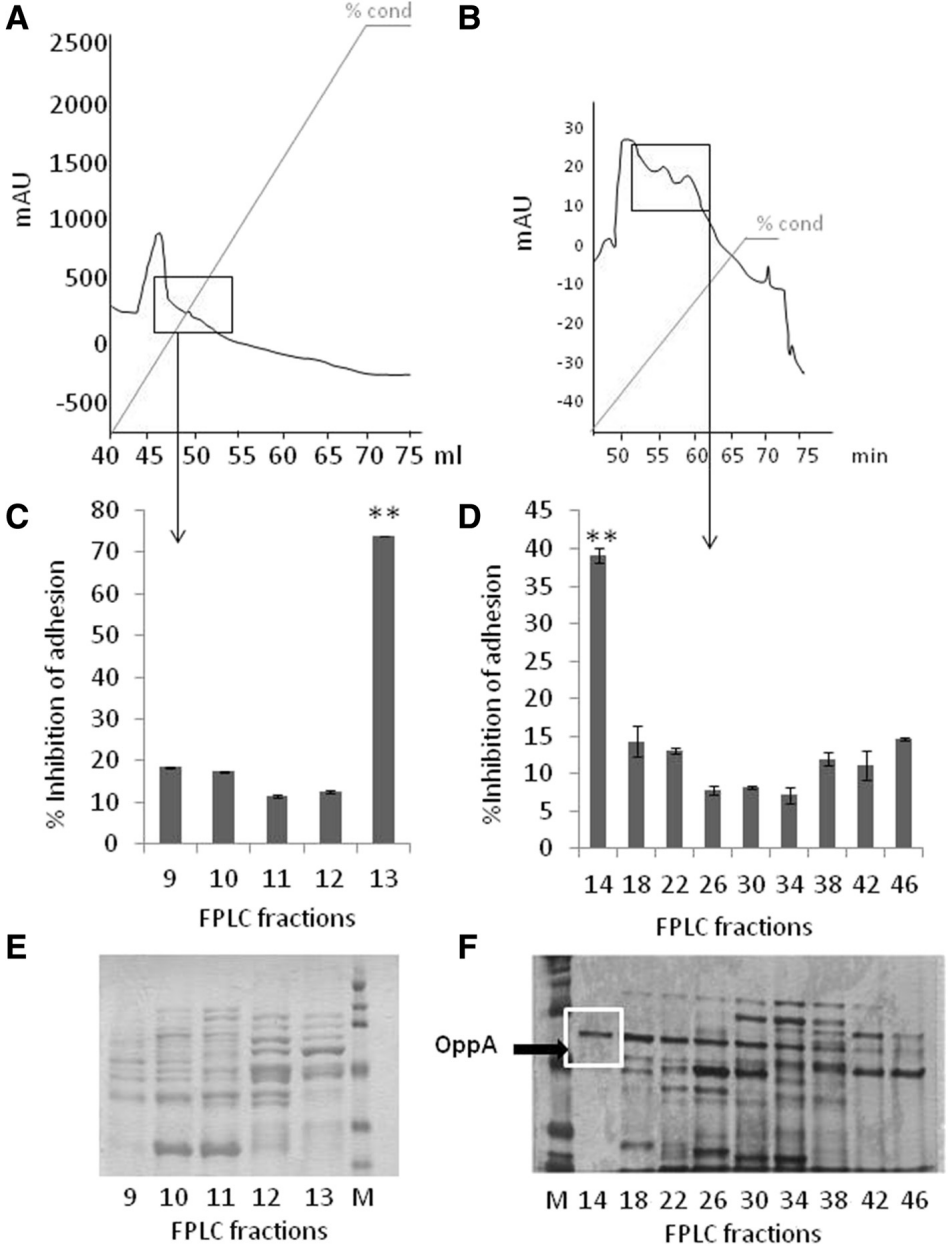
**Surface proteins of *Lactobacillus salivarius *Lv72 separated by means of heparin-affinity chromatography (A, C, E) and ionic interchange chromatograpy (B, D, F). A**,**B)** Chromatograms. The mark shows the fractions of interest that were tested further. **C**,**D)** Adherence interference experiments: inhibitory effect of the fractions on Lv72 binding to HeLa cells. **E**,**F)** SDS-PAGE of the isolated fractions. n=6 ANOVA test **, p-value < 0.001.

**Figure 5:**
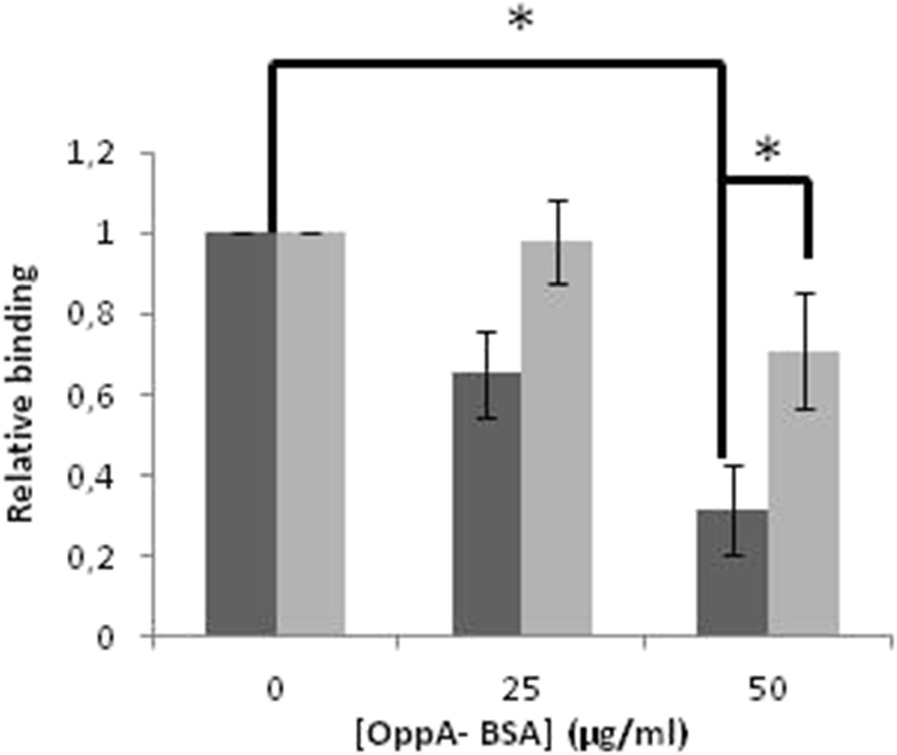
**Inhibition of *L. salivarius *Lv72 attachment to HeLa cells by different concentrations of purified OppA.** Lv72 was co-incubated in the presence of OppA (■) or bovine serum albumin (used as a negative control) (grey sqaure). n=5 ANOVA test *, p-value < 0.05.

To identify the bacterial proteins involved in the interaction between *L. salivarius* Lv72 and eukaryotic GAGs, the proteins of the bacterial envelope were solubilised and subjected to affinity chromatography, using heparin as the ligand. The fractions eluting at concentrations higher than 0.8 M NaCl were tested for their ability to interfere with the HeLa – *L. salivarius* binding. Those showing high activity were subjected to anion exchange chromatography. One of the fractions recovered showed a high interfering activity while presenting just one conspicuous protein band upon SDS-PAGE analysis (Figure 4). This protein was identified by MALDI-TOF (MS) analysis as a soluble binding protein of an ABC transport system due to its homology with the protein OppA of *Lactobacillus salivarius* UCC118 (GI/90962668) (9 queries matched, 10% sequence coverage). The gene encoding for *L. salivarius* Lv72 OppA was cloned in *E. coli*, overexpressed and purified by passage through a heparin affinity column. The purified protein was used in interference adhesion assays (Figure 5). The results obtained show that OppA significantly interferes with the attachment of *L. salivarius* Lv72 to HeLa cultures in a dose dependent way, thus confirming its role as an adhesin in the interaction between both cellular types.

## Discussion

PGs are ubiquitous, being present in all cell types and in the ECM. The enormous structural diversity of their GAG chains and core proteins mediates specific interactions between many molecules. Because of these characteristics, they play an essential role in the interaction between cells. In addition, these molecules present properties which suggest that they might be part of the receptors that allow the attachment of the normal microbiota to the mucous epithelia that line the digestive tract and the vagina. In fact, many pathogenic microorganisms use these molecules as specific receptors and in bacterial internalization during the infective process [39,40].

### The GAGs are involved on HeLa cell-*Lactobacillus salivarius* Lv72 adhesion

To test the probiotic bacteria-PG receptor hypothesis, a model was devised that involved HeLa cells, a line derived from human genital epithelium, and a vaginal strain of *Lactobacillus salivarius*, which was previously shown to adhere to HeLa cells [1]. However, caution should be taken when interpreting these results, as HeLa cell line has been found to be unstable and its gene expression profiles differ from those in normal human tissues [41].

The experiments involved the GAGs HS, CS A, and CS C, usually present on the cell surface as part of PGs such as syndecans, glypicans, betaglycan or different isoforms of CD44. Heparin (an oversulfated form of HS) and CS B (DS) were also included in the studies. The results indicate that all these GAGs with the exception of CS B were able to efficiently interfere with *L. salivarius* binding, the effect ranging between 50% and 60% for heparin and CS A and C respectively. Their combined effects were nearly additive, the mixture of all species rising to 90% inhibition of the bacterial binding. These data were confirmed by the observation that enzymatic elimination of surface GAGs resulted in blockage of *L. salivarius* attachment to the HeLa cell cultures.

However, residual attachment always remained after GAG interference or digestion suggesting that other eukaryotic receptors may be involved. In fact, cell-associated ECM proteins such as fibronectin, laminin and collagen have been identified as receptors, especially for pathogenic bacteria [42-44] and also for vaginal and intestinal lactobacilli [45,46]. In addition, direct binding between lactobacilli and glycolipids of the epithelial cell membranes appear to contribute to the attachment, in a process mediated by divalent cations [47]. Finally, non-specific factors might also contribute to cell to cell adherence, especially superficial hydrophobicity established between membrane exposed patches of the eukaryotic cell and components of the Gram positive cell wall, especially teichoic acids [48].

### *L. salivarius* Lv 72 has different affinity for the different GAGs

**Figure 6:**
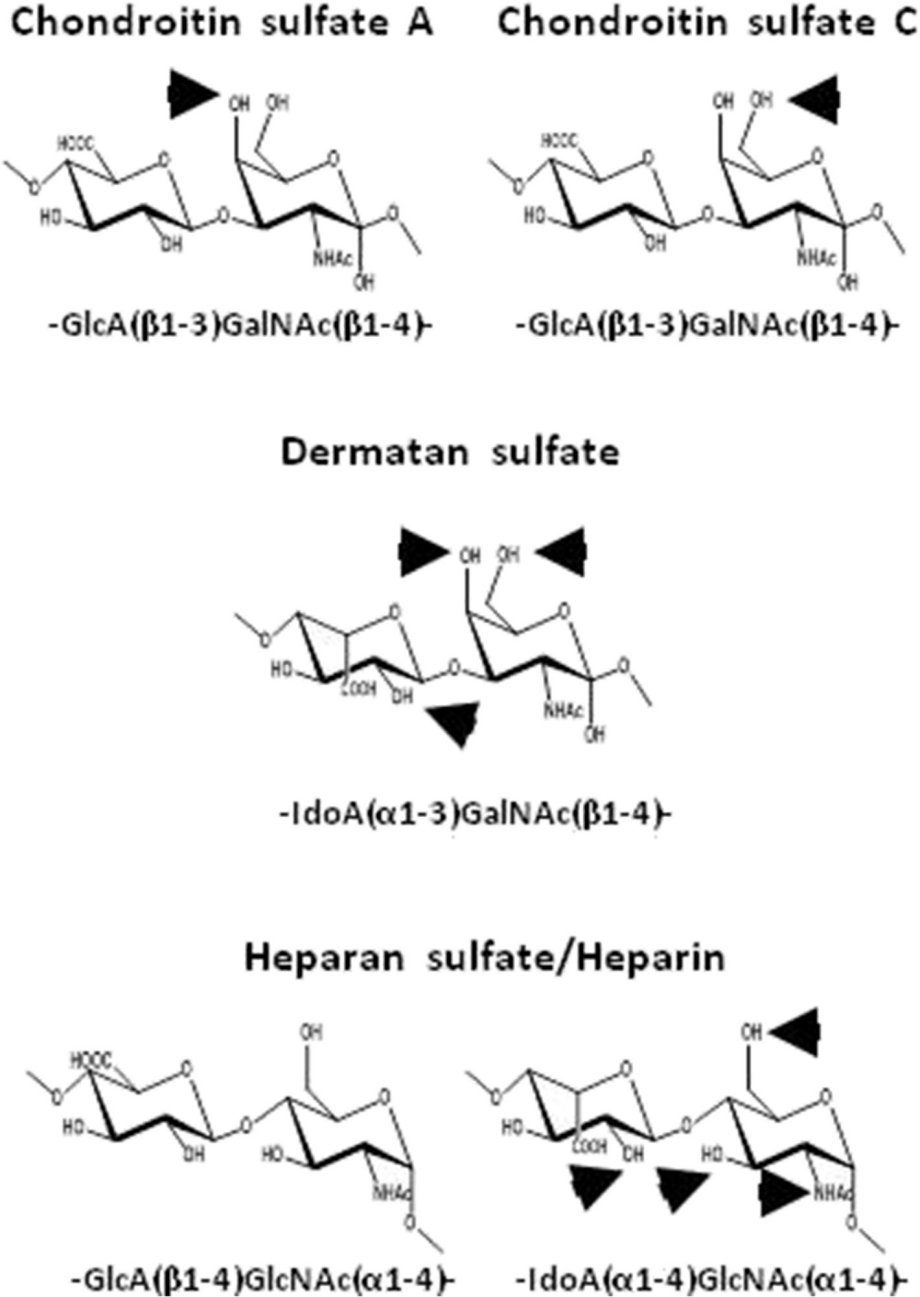
**Disaccharide units of GAGs: CS A is sulfated at C4 of GalNAc (pointed by an arrow).** CS C is sulfated at C6 of GalNAc (pointed by an arrow). In CS B (DS) GlcA is epimerized to IdoA, and can be sulfated at C4 or C6 of GalNAc and C2 of IdoA. HS includes GlcA and IdoA residues and can be sulfated at C2 of the uronic acid residue and at N, C6 and C3 of GlcN; heparin is basically constituted of IdoA-GlcN oversulfated disaccharides.

In spite of the general effect of GAGs on bacterial attachment, different molecules displayed apparent disparate interference constants. Among the group of CSs, characterized by being composed of uronic acid linked to the third carbon of N-acetylgalactosamine, CS C appears to be 6 times more active than CS A. Conversely, CS B generated a binding increase. This might be due to the different sulfation patterns shown; CS A and C are sulfated at C-4 and C-6 of the GalNAc moieties respectively, while CS B is usually more extensively sulfated (Figure 6). Additionally, the GlcA residue present in CS A and C is epimerized to IdoA in CS B, which confers greater conformational flexibility on the molecule [49]. The glucosaminoglycans are represented by HS and heparin and are composed of uronic acid linked to the fourth carbon of glucosamine. In spite of their fundamental similarity, heparin displays an apparent affinity that is lower than that of HS. The main difference between these two molecules is that HS displays IdoA rich regions of very high sulfation that can extend from two to eight disaccharide units and are separated by 15 poorly sulfated GlcA rich disaccharide moieties [50]. Heparin, on the other hand, shows more extensive sulfation and uronic acid epimerization (Figure 6). Taken together, these data indicate that the regiochemistry of the sulfation is crucial for affinity of the binding as evidenced by the difference between the CS sulfated at C-4 or C-6, or the significant difference between the oversulfated heparin and the HS. Furthermore, the epimerization of the uronic acid seems also to be crucial, based on the difference in behavior induced by IdoA-rich species, such as heparin and, particularly, CS B.

The high affinity of particular bacteria for HS and heparin has been observed with several pathogens. For instance, both molecules bind strongly to *Pneumococci*, *Penicillium*, *Enterococci* and *Listeria*[25,51-53]. Conversely, heparin displays greater affinity for *Chlamydia*[54] while HS does so for *Pseudomonas*[55]. The CSs are high affinity receptors for *Pneumococci*[53] or *Spirochetes*[56] although they do not bind to *Chlamydia*, *Penicillium*, *Pseudomonas* or *Listeria*[51,52,54,55]. Interestingly, DS usually shows a different behavior compared to other molecular forms of galactosaminoglycans, acting as receptor in *Chlamydia*, *Penicillium* or *Leptospira*[52,54,57], although, to our knowledge, this is the first communication on an increase of bacterial binding in the presence of this molecule in solution.

### The GAGs obtained from different cell types have different effect on adherence

The fine structure of the GAGs differs according not only to their nature, but also to the developmental phase and the physiological and pathological conditions as well as to the cellular type. This is especially noticeable for HS, but also for CS/DS [50,58,59]. GAGs isolated from HeLa and HT-29 cells notably increased the inhibition of binding in comparison to the commercial forms, which were isolated from bovine kidney (HS), bovine trachea (CS A), shark cartilage (CS C) and porcine mucosa (CS B).

### OppA protein is an adhesin involved in Lv 72 adhesion to HeLa cells

Once the nature of the main eukaryotic cell receptors was known, identification of bacterial adhesins became easier because the prior could be employed as affinity ligands for the latter. In this way, using heparin as ligand, we identified OppA, which strongly interfered with HeLa – *L. salivarius* attachment in a concentration dependent manner. However, a plateau was reached which suggests that other adhesins may exist and that they recognize non-overlapping receptors as happens with *Borrelia burgdorferi*, which presents 4 adhesins capable of GAG binding [56].

OppA is an externally exposed extracellular lipoprotein carrying a peptidase II signal for covalent anchoring to the membrane [12] to which diverse roles have been ascribed; it acts as the substrate-binding protein of the oligopeptide transport system in lactobacilli [60], but has also been implicated in cytoadhesion of *Mycobacterium hominis* and *Treponema denticolaria* to eukaryotic cells through interaction with plasminogen and fibronectin respectively [61-63]. Furthermore, it has been found to interact with fibronectin and collagen in *Lactobacillus casei* BL23 and other OppA orthologues from lactobacilli such as MapA from *L. reuteri* are able to interact with Caco-2 [12,64]. The OppA-mediated cytoadhesion of *M. hominis* to HeLa cells seems to be dependent on the ATPase activity of the protein [63,65]. These precedents and the data reported here on adhesion to GAGs, indicate that OppA is a multifunctional protein that mediates the interaction of the bacteria with its environment. Attachment to the substrate may be a means of accessing peptides that will subsequently be internalized [66], especially for multiauxotrophic organisms such as the lactobacilli.

## Conclusion

In conclusion, the adherence of *L. salivarius* Lv72 to HeLa cells is, at least in part, mediated by the interaction of the bacterial OppA protein and the GAGs present on the eukaryotic surface.

## Methods

### Bacterial strains, eukaryotic cell lines and growth conditions

*Lactobacillus salivarius* Lv72 (CECT 8259) (*Colección Española de Cultivos Tipo* (CECT), Valencia, Spain) was isolated from the vaginal fluid of a healthy woman of reproductive age [1]. It was propagated in MRS broth (Becton, Franklin lakes, USA) set at 37°C without agitation in a 10% (v/v) CO_2_ enriched atmosphere. When appropriate, 1.5% (w/v) agar was added to the liquid medium.

HeLa (ATCC CCL-2) and HT-29 (ATCC HTB-38) cell lines (LGC-Standards, Molsheim, France) were grown in Dulbecco’s Modified Eagle’s minimal essential medium (DMEM) (GibcoBRL, Eragny, France) supplemented with 10% (w/v) fetal bovine serum (GibcoBRL) and with penicillin G/streptomycin (5000 IU/ml, 5000 μg/ml) (Sigma-Aldrich Chemie GmbH, Buchs, Switzerland). Cultures were incubated in 25 cm^2^ tissue culture flasks (Nunc, Roskilde, Denmark) at 37°C in a 5% (v/v) CO_2_ atmosphere until confluence. For adhesion assays, 2000 cells per well were seeded in 24-well culture plates (Nunc) and cultivated, with a daily change of the culture medium until confluence.

### Fluorescein labeling

Fluorescein isothiocyanate (FITC) (Sigma-Aldrich) labeling was performed on overnight cultures washed four times with PBS buffer (GibcoBRL) and resuspended in a 0.1 mg/ml FITC solution to an A_600_ of 0.5; incubation in the dark at 37°C under agitation proceeded for 2 h and the bacterial suspensions were centrifuged and washed 4 times with PBS to eliminate the excess FITC.

### Adherence assays

Adhesion of *L. salivarius* Lv72 to HeLa monolayers was tested following the procedure described by Tallon and co-workers using 25 FITC-labelled bacteria per eukaryotic cell [67]. At the end of the experiment, epithelial cells were disaggregated with trypsin and the fluorescence of the lactobacilli attached to them was quantified in a Perkin Elmer LS55 fluorometer set at 488 nm (excitation) and 560 nm (emission). Data were normalized using the adhesion values without any additive which was given the arbitrary value of 1. Assays were performed at least in triplicate and the data are expressed as the mean ± SD.

Adherence interference experiments were performed with heparin, HS, CS A, CS B (DS) and CS C (Sigma-Aldrich) and their combinations at concentrations ranging between 0.01 and 100 μg/ml (final concentration), added to the monolayers immediately before the bacterial cultures. Complementarily, the surface GAGs of HeLa and HT-29 cells and the bacterial surface proteins of *L. salivarius* Lv72 as well as OppA were extracted and purified (see below) and also used in adherence interference experiments. The dissociation constant estimations were obtained through a non-linear regression using the program Statistica (StatSoft, Inc. USA) by means of the equation of Langmuir [68].

### Enzymatic digestion of eukaryotic cell-surface GAGs

Hydrolysis of HS from cell cultures was achieved by overnight incubation at 37°C in DMEM minimal medium with a mix of 500 mU/ml (final concentration) of each heparinases I, II and III (Sigma-Aldrich). Elimination of CS/DS was obtained through incubation of the cell cultures with 250 mU/ml of chondroitinase ABC (Sigma-Aldrich) at 37°C for 3 h. Elimination of both GAGs was achieved through successive incubation of the cell cultures with the enzymatic mixes, with an intermediate washing with PBS buffer. The reactions were stopped with 2 washes in PBS buffer and the cell cultures were immediately submerged in DMEM and subjected to adherence assays with *L. salivarius* Lv72 as described in a previous paragraph.

### GAG extraction and purification

HeLa and HT-29 cells were propagated in 10 cm diameter tissue plates (Nunc) until confluence. The monolayers were washed twice with PBS and incubated in 6 ml of a 6 M guanidinium chloride, 3 mM DTT (Sigma-Aldrich) solution in 50 mM Tris–HCl (pH 8) at 60°C for 1 h with agitation. Afterwards, 15 ml of a 6.7 mM CaCl_2_ (Merck, Lion, France) solution in Tris–HCl (pH 8) plus 1.5 μg/ml proteinase K (Sigma-Aldrich) were added and the culture supernatant was recovered and incubated overnight at 56°C. Subsequently, the proteinase K was inactivated by incubation at 100°C for 10 min; 5.7 volumes of ethanol (VWR) were added followed by incubation at 4°C for 2 h. The precipitated GAGs were pelleted at 4000 x g for 15 min, air-dried and resuspended in 1 ml of a 0.2 M NaOH, 0.8% sodium borohydride (Merck) solution and agitated overnight at room temperature. The resulting suspension was centrifuged at 12,000 x g and the GAGs present in the supernatant were precipitated with ethanol (85%), dried and resuspended in 1 ml distilled water. The GAG concentration was determined spectrophotometrically as described previously [69]. The partial digestion of HS and CS was performed as described above.

### Extraction of *L. salivarius* Lv72 surface proteins and heparin-affinity chromatography

*L. salivarius* Lv72 was grown until mid-exponential phase, washed twice with buffer A (50 mM Tris–HCl, 150 mM NaCl; pH 7.5) and the bacterial cell pellet was resuspended in the same buffer containing a commercial cocktail of EDTA-free protease inhibitors (Roche, Basel, Switzerland), 1 mM MgCl_2_, 5 mg/ml lysozyme (Sigma-Aldrich) and 0.05 U/ml mutanolysin (Sigma-Aldrich) and incubated overnight at 4°C. Cells were mechanically disrupted by repeated passage through a French press (SLM Aminco Inc), the pellet was washed twice with buffer A and subjected to overnight digestion with 5 mg/ml lysozyme in the presence of protease inhibitors at 4°C, followed by incubation with 5% Triton X-100 (Sigma-Aldrich) for 1 h at room temperature. The final solution was centrifugated at 10,000 rpm for 30 min and the supernatant was applied to a 1 ml heparin affinity column (GE, Buckinghamshire, England) connected to a FPLC system (GE). Bound proteins were eluted with a continuous 0 – 2 M NaCl gradient in 50 mM Tris–HCl buffer (pH 7.5) and aliquots of the protein fractions were used in HeLa/*Lactobacillus* adherence assays. Those that interfered most were subjected to anion exchange chromatography in a Q-sepharose FF column (GE), eluted with a continuous 0 – 0.5 M NaCl gradient in 50 mM Tris–HCl buffer (pH 7.5) and the resulting fractions were subjected to adherence interference assays as described above. The protein concentrations were determined with the Pierce BCA Protein Assay Kit (Thermo Scientific, Rockford, USA) following the instructions of the manufacturer. SDS-PAGE [66] was performed in a “Miniprotean III” system (Bio-Rad, Hercules, USA). The proteins were stained with Comassie R-250 blue [70] or with a protein silver staining kit (GE). The band of interest was excised from the gels, digested with porcine trypsin and the resulting peptides were analyzed by MALDI-TOF/(MS) at the Proteomic Service of the *Centro Nacional de Biotecnología* (CNB-CSIC, Madrid).

### Construction of expression plasmids and purification of the oligopeptide permease A protein (OppA)

The *oppA* sequence of *L. salivarius* Lv72 [BankIt1609288 Lactobacillus KC703973] was amplified using primer pairs deduced from the *oppA* sequence of *L. salivarius* UCC118 (LSL_1882). The sequence encoding the OppA signal peptide was omitted to ease protein purification. The PCR product was purified and cloned into the vector pRSET-B digested with *Nde*I and *Bam*HI (Fermentas, Thermo Scientific). The resulting plasmid was transformed to *E. coli* BL21 pLys (DE3)/pLysS cells, which were grown to an OD_600_ of 0.6 at 37°C with shaking before addition of 1 mM IPTG (Fermentas, Thermo Scientific) and incubation was continued at 28°C with shaking overnight. The cultures were harvested, resuspended in 25 mM Tris–HCl (pH 7.5) containing 0.05% Triton-X100 and disrupted by sonication. The supernatant proteins were fractionated after passage through a heparin-affinity chromatography column as described above and the purified OppA protein was used for adhesion assays at concentrations ranging from 1 to 50 μg/ml.

### Statistical analysis

Statistical analysis was performed using GraphPad Prism Software version 5.00 for Windows (San Diego, California, USA). The groups were compared using one-way analysis of variance (ANOVA) followed by the student-Newman-Keuls multiple comparison post hoc analysis. A p-value of less than 0.05 was considered significant.

## Competing interests

The authors declare that they have no competing interests.

## Authors’ contributions

RM carried out the adhesion assays, the enzymatic treatments and the isolation and identification of OppA protein and drafted the manuscript. CM participated in GAGs extraction and in the adhesion assays. SM carried out the clonage and purification of the OppA protein. ES and LQ conceived the study and participated in its design and coordination and helped to draft the manuscript. All authors read and approved the final manuscript.
